# Analysis of current family management style and influencing factors in young children with bronchial asthma

**DOI:** 10.3389/fped.2026.1724582

**Published:** 2026-03-05

**Authors:** Yingying Ye, Yi Wang, Hong-Zhen Xu

**Affiliations:** 1 School of Nursing, Zhejiang Chinese Medical University, Hangzhou, China; 2 Department of Nursing, Children's Hospital, Zhejiang University School of Medicine, National Clinical Research Center for Child Health, Hangzhou, China

**Keywords:** asthma, factor, management styles, nurse, pediatric

## Abstract

**Objective:**

Investigate the current status of family management styles among families of children aged 1–7 years with bronchial asthma and analyze influencing factors to provide a basis for developing intervention strategies.

**Methods:**

A cross-sectional questionnaire survey was administered between February and December 2024 to 257 pediatric patients with physician-diagnosed asthma and their primary caregivers at a tertiary care children's hospital in Zhejiang, China. The family management styles of children with asthma and their influencing factors were assessed using a general information questionnaire, the Childhood Asthma Control Test (C-ACT, for children aged five and above), the Test for Respiratory and Asthma Control in Kids (TRACK, for children under five), and the Family Management Scale for Children with Asthma.

**Results:**

The Family Management Scale for Children with Asthma scores were (239.30 ± 20.38) points, above average. Univariate analysis revealed that the child's nutritional status and the caregiver's educational level significantly influence asthma management styles (*P* < 0.05). Multiple linear regression analysis found child's wasting was associated with higher FMSCA scores (Beta = 0.16, *P* < 0.05), indicating better management styles. However, child's obesity was associated with lower scores (Beta = −0.13, *P* < 0.05), indicating worse management styles. Regarding educational level, caregivers with junior high school education or below were associated with lower scores (Beta = −0.19, *P* < 0.05) compared to those with junior college qualifications, indicating worse management styles.

**Conclusion:**

The family management style for pediatric asthma patients (children aged 1–7 years) was adaptive and primarily influenced by the child's nutritional status and the caregiver's level of education. Specifically, child's obesity and a caregiver education level of junior high school or below were associated with worse family management styles, whereas child's wasting was linked to better family management styles.

## 1 Introduction

Bronchial asthma (here in after referred to as asthma) is one of the most common chronic respiratory diseases affecting children globally and poses a significant threat to their health and quality of life. Recent studies have indicated that the global prevalence of childhood asthma is approximately 10.2% (1). In the United States, it affects over 5 million children annually and is associated with approximately 200 deaths annually (2). In mainland China, the prevalence of asthma among children aged 0–14 is 2.27%, a figure that has been steadily increasing (3). Notably, the prevalence among children aged 1–7 years is approximately 3.27% (4), which is higher than that in other age groups (2.52%) (5). Alarmingly, the proportion of uncontrolled asthma among Chinese children is the highest in the Asia-Pacific region (6), severely impacting their physical growth, psychological well-being, and family quality of life (7). Although pharmacotherapy is key to symptom control, asthma management predominantly occurs at home. Effective family management, encompassing symptom monitoring, environmental control, and medication adherence, is crucial for achieving optimal disease outcomes.

Family management styles are a crucial concept in chronic illness care, referring to the distinctive patterns of response that a family adopts in managing a child's condition (8). These styles are commonly categorized into five types: thriving, adaptive, enduring, struggling, and floundering (9). They form a continuum ranging from “well-managed” to “challenged.” Among them, the thriving management style involves normalizing the illness and proactively managing it with confidence. The adaptive style describes families who function largely normally but must navigate additional challenges and negative emotions. In the enduring style, families perceive the illness as a heavy burden, striving to manage it yet feeling exhausted. The core issue for struggling families lies in severe parental conflict and internal friction over management approaches. Floundering families, meanwhile, become mired in chaos and uncertainty, feeling bewildered and unable to respond effectively. An effective family management style is closely associated with improved asthma control, better lung function, and enhanced family functioning (10). However, family management style varies substantially across families and is influenced by factors such as the child's illness severity, parental health literacy, and access to healthcare resources (11). For children aged 1–7 years, whose cognitive development is incomplete, disease control is highly dependent on family management (12). Nevertheless, the predominant styles of family management and their key determinants within this specific age group have not been fully elucidated yet.

Current research on family management styles has primarily focused on conditions such as leukemia (13) and Prader-Willi syndrome (14), whereas systematic investigations of young children with asthma remain limited. Importantly, evidence indicates that the initial symptoms of asthma in many adolescents can be traced back to early childhood (5), highlighting the critical role of scientific management during these formative years in shaping the long-term outcomes of the disease. Therefore, this study aimed to investigate the current status of family management styles and analyze their associations with both child-related and caregiver-related factors with the goal of providing an evidence-based foundation for developing targeted clinical interventions.

## 2 Material and methods

### 2.1 Study design and participation

This study employed a cross-sectional survey design. Between February and December 2024, trained investigators used convenience sampling to consecutively recruit all eligible pediatric asthma patients and their caregivers meeting inclusion and exclusion criteria at the respiratory outpatient clinic of a tertiary children's specialty hospital in Zhejiang, China. Inclusion criteria and Exclusion criteria are as follows.

Inclusion criteria for children:

(1) Diagnosed with pediatric bronchial asthma according to the Guidelines for the Prevention and Management of Bronchial Asthma (2016 Edition) (15);
(2) Aged between 12 and 83 months;
(3) Diagnosis established at least one month prior to enrollment.

Exclusion criteria for Children:

If they had been diagnosed with any other chronic medical condition within the month prior to enrollment, including but not limited to nephrotic syndrome, leukemia, congenital heart diseases, or acute severe respiratory infections.

Inclusion criteria for caregivers:

(1) Possession of normal communication, reading, and writing abilities;
(2) Co-residing with the patient and serving as the primary, non-remunerated caregiver responsible for the child's daily care activities.

Exclusion criteria for caregivers:

(1) Experience of a major life event (e.g., divorce, death of a significant relative) within the past three months.;
(2) Diagnosis of a severe chronic illness (e.g., uremia).

### 2.2 Sample size calculation

In multivariate analysis, the sample size should be at least 5–10 times the number of study variables (16, 17). This study includes 20 variables. Considering a 20% dropout rate, the estimated required sample size ranges from 120 to 240 cases.

### 2.3 Demographic information of survey participants

General information was compiled by researchers through literature review and discussions with clinical asthma specialists, encompassing two sections: pediatric patients and caregivers. The pediatric section includes: child's age (in years), gender (male/female), disease course (in months), desensitization therapy (yes/no), only-child status (yes/no), gestational age (in weeks), mode of delivery (vaginal delivery/cesarean section), feeding practices during the first 6 months of life (exclusive breastfeeding/formula feeding/mixed feeding), body mass index (severe wasting/wasting/normal/overweight/obese/severe obesity), school enrolment (not yet enrolled in school/kindergarten/primary school), residence and family history (yes/no). The caregiver section includes: caregiver's role (mother/father/paternal grandmother/maternal grandmother/maternal grandfather/ paternal grandfather), age (in years), educational level (junior high school or below/middle school/junior college/undergraduate/postgraduate), marital status (married/divorced/widowed), type of family (nuclear family/stem family/step family/other), annual income (in RMB), work-related changes attributable to disease (yes/no), etc.

When calculating nutritional status, note that BMI based on weight and length applies to children under 2 years old, while BMI based on weight and height applies to those aged 2 to under 7 years. Age is recorded in full months or full years. Weight, height, or length is measured by hospital outpatient nurses, and BMI is reported according to pediatric growth reference standards (18).

### 2.4 Test for respiratory and asthma control in kids, TRACK

TRACK, originally developed by Murphy et al. (19) and later culturally adapted by Hong (20), is a tool designed to evaluate asthma control in children under the age of five over the preceding four weeks. The instrument comprises five items: limitations in activity, dyspnea, occurrence of cough symptoms, utilization of emergency medications, and a self-assessment of asthma control during the past month. Each item offers five response options, with scores ranging from 0 to 20 points, culminating in a total score that spans from 0 to 100 points. The TRACK score of 80 or above signifies good asthma control, scores between 60 and 79 indicate partial control, and scores below 60 reflect poor asthma control (21). The Cronbach's α coefficient for the Chinese version of the scale was reported at 0.704, demonstrating satisfactory reliability and validity (22). The study used TRACK to assess asthma control levels in children aged 1–4 years.

### 2.5 Children-asthma control test, C-ACT

ACT, developed by Liu et al. (23), evaluates asthma control in 4–11 year-old children during the preceding four weeks. The scale consists of seven items. The first four items have four options each, with scores ranging from 0 to 3 points; the last three items have six options each, with scores ranging from 0 to 5 points, for a total score of 0–27 points. The C-ACT score of 19 or less indicates poor asthma control, 20–23 points indicate partial control, and a score of 24 or higher indicates complete control (24). The Cronbach's *α* value was 0.80, indicating good reliability and validity (24). The study utilized the scale to assess asthma control levels in pediatric patients aged 5−7 years.

### 2.6 The family management scale for children with asthma, FMSCA

FMSCA was developed by Duan's research group (25). This scale includes 57 items distributed across eight dimensions, namely, Children Identity, View of Condition, Management Mindset, Parental Mutuality, Parenting Philosophy, Management Approach, Family Focus, and Future Expectation. Each item is evaluated using a 5-point Likert scale, where scores range from 1 to 5, reflecting a continuum from complete disagreement to complete agreement. A higher total score indicating a better level of family management. The Cronbach's α of 0.918, with good reliability and validity. Consequently, it serves as a valid instrument for assessing the family management levels of Chinese children with asthma (25).

### 2.7 Data collection and quality control

The survey was conducted by uniformly trained researchers in quiet areas of outpatient clinics. After obtaining informed consent, questionnaires were distributed to caregivers via the Questionnaire Star platform, with on-site explanations of completion requirements. For items that posed comprehension difficulties, investigators provided explanations in standardized, neutral language. All information was completed by caregivers, except for the first four questions of the C-ACT asthma control questionnaire for children aged 5–7 years, which were answered by the children themselves. Completed questionnaires were collected immediately and checked for completeness. Data were entered independently by two separate individuals.

### 2.8 Ethics committee approval

The study was approved by the Medical Ethics Committee of Children's Hospital, Zhejiang University School of Medicine, (NO. 2024-IRB-0027-P-01).

### 2.9 Statistical analysis

Statistical analysis was performed using SPSS26.0 software. Descriptive statistics were performed on the total scores and dimension-specific scores of the FMSCA. For continuous variables, means ± standard deviations were reported if data met normality assumptions, assessed using the Shapiro–Wilk test. Otherwise, medians (interquartile ranges) were used. Categorical variables were described using frequencies (percentages). Univariate analyses were conducted to examine differences in FMSCA total scores across different categories of socio-demographic characteristics and asthma control status. Methods included *t*-tests or analysis of variance (ANOVA). For categorical variables that showed statistical significance in the ANOVA, further *post-hoc* pairwise comparisons were conducted. First, the homogeneity of variances across groups was assessed using Levene's test. If the assumption of homogeneity of variances was met (*P* > 0.05), Tukey's HSD (Honestly Significant Difference) method was employed for *post-hoc* comparisons. If the assumption was violated *(P* < 0.05), the Games-Howell test was used.

Subsequently, variables that were statistically significant in univariate analyses were used as independent variables. Using the total FMSCA score as the dependent variable, multiple linear regression (stepwise) was employed to analyze the independent variables. Categorical predictors with more than two levels were entered as dummy variables. Reference values are established based on clinical significance and research objectives. Statistical hypothesis testing: Prior to regression analysis, test for residual independence, homogeneity of variance, and diagnose multicollinearity among independent variables. In all statistical analyses, *P* < 0.05 was considered statistically significant.

## 3 Result

### 3.1 Demographic and clinical characteristics

A total of 260 questionnaires were distributed in this study, yielding 257 valid responses, for a response rate of 98.85%. Among these participants, 127 were toddlers and preschoolers (aged 1–4 years), and 130 were school-aged children (aged 5–7 years). Among children, 70.4% were male and 29.6% female. The study found that the majority of caregivers were mothers, accounting for 83.7% of the sample. The marital status of caregivers was predominantly married (*n* = 255, accounting for 99.2%), with only one case each of divorced and widowed individuals. TRACK and C-ACT measurements showed that only 102 children (39.69%) achieved complete control, 94 children (36.58%) were partially controlled, and 61 children (23.74%) remained uncontrolled. The uncontrolled state was most prevalent among children aged 5–7 years, with 32 cases accounting for 52.46%. Detailed information is presented in Tables 1, 2.

**Table 1:** Demographic information, clinical characteristics and univariate analysis of FMSCA in children (*n*=257).

Demographic variables		*N*	Percent (%)	FMSCA (x ± s)	t/F	Effect size (95%CI)	*P*
Age (year)	1–4	127	49.40	239.14 ± 18.55	−0.12	−0.010 (−0.260, 0.240)	0.905
	5–7	130	50.60	239.45 ± 22.10			
Gender	Male	181	70.40	238.59 ± 21.04	−0.86	−0.120 (−0.390, 0.150)	0.393
	Female	76	29.60	240.97 ± 18.75			
Disease cource (month)	1–3	50	19.50	239.18 ± 18.63	0.51	0.014 (0.000, 0.035)	0.824
	4–6	35	13.60	238.43 ± 20.15			
	7–12	46	17.90	242.89 ± 19.17			
	13–24	56	21.80	238.00 ± 20.14			
	25–36	39	15.20	240.28 ± 24.83			
	37–48	22	8.60	235.82 ± 20.64			
	49–60	7	2.70	233.43 ± 21.54			
	>60	2	0.80	250.50 ± 0.71			
Desensitizat-ion therapy	Yes	14	5.40	241.57 ± 15.01	0.43	0.120 (−0.420, 0.660)	0.668
	No	243	94.60	239.16 ± 20.66			
Only-child status	Yes	134	52.10	240.84 ± 19.32	1.27	0.160 (−0.090, 0.410)	0.204
	No	123	47.90	237.61 ± 21.42			
Gestational age (Week)	<37	21	8.17	240.19 ± 17.89	0.42	0.003 (0.000, 0.015)	0.658
	37–42	224	87.16	238.94 ± 20.98			
	>42	12	4.67	244.33 ± 11.44			
Mode of delivery	Vaginal delivery	151	58.75	239.50 ± 21.03	0.19	0.020 (−0.230, 0.270)	0.851
	Cesarean section	106	41.25	239.01 ± 19.52			
Feeding practices during the first 6 months of life	Exclusive breastfeeding	149	58.00	237.66 ± 21.75	1.20	0.009 (0.000, 0.030)	0.302
	Formula feeding	25	9.70	242.84 ± 18.51			
	Mixed feeding	83	32.30	241.16 ± 18.17			
Body mass index	Severe wasting	3	1.20	245.33 ± 16.26	3.12	0.059 (0.010, 0.112)	0.009
	Wasting	18	7.00	252.22 ± 16.38			
	Normal	195	75.90	238.18 ± 19.46			
	Overweight	24	9.30	243.83 ± 19.00			
	Obese	14	5.40	227.07 ± 31.42			
	Severe obesity	3	1.20	249.00 ± 14.93			
School enrolment	Not yet enrolled in school	29	11.30	237.86 ± 17.01	0.43	0.003 (0.000, 0.015)	0.648
	Kindergarten	179	69.60	238.88 ± 20.71			
	Primary School	49	19.10	241.65 ± 21.19			
Residence	City	160	62.30	239.85 ± 19.51	0.81	0.006 (0.000, 0.025)	0.445
	Town	75	29.20	239.67 ± 22.29			
	Rural	22	8.60	234.00 ± 19.95			
Family history	Yes	214	83.30	239.38 ± 20.70	0.15	−0.030 (−0.360, 0.300)	0.878
	No	43	16.70	238.86 ± 18.91			
Asthma control status	Controlled	102	39.69	242.54 ± 18.43	2.67	0.023 (0.000, 0.055)	0.073
	Partial controlled	94	36.58	234.57 ± 24.67			
	Uncontrolled	61	23.73	238.84 ± 15.81			

A BMI between +1 SD and −2 SD is considered normal; BMI ≥ +1 SD and < +2 SD is classified as overweight; BMI ≥ +2 SD and < +3 SD is classified as obese; BMI ≥ +3 SD is classified as severe obesity; BMI ≥ −3 SD and < −2 SD is considered wasting; BMI < −3 SD is considered severe wasting.

**Table 2:** Demographic information, clinical characteristics and univariate analysis of FMSCA in caregivers (*n*=257).

Demographic variables (*n*, %)		*N*	Percent (%)	FMSCA (x ± s)	t/F	Effect Size (95%CI)	*P*
Role	Mother	215	83.70	238.59 ± 21.12	1.28	0.020 (0.000, 0.050)	0.279
	Father	24	9.30	245.13 ± 14.24			
	Paternal grandmother	12	4.70	234.58 ± 16.84			
	Maternal grandmother	5	1.90	248.80 ± 15.88			
	Paternal grandfather	1	0.40	–			
	Maternal grandfather	0	0.00	–			
Age (years)	20–29	32	12.50	237.22 ± 3.79	0.57	0.009 (0.000, 0.030)	0.685
	30–39	179	69.60	238.92 ± 1.58			
	40–49	28	10.90	243.32 ± 3.09			
	50–59	14	5.40	238.29 ± 4.62			
	≥60	4	1.60	248.25 ± 8.62			
Educational level	Junior high school or below	26	10.10	227.35 ± 23.02	3.10	0.047 (0.005, 0.095)	0.016
	Middle school	30	11.70	238.33 ± 19.65			
	Junior college	69	26.80	241.12 ± 18.30			
	Undergraduate	118	45.90	241.69 ± 20.42			
	Postgraduate	14	5.40	234.36 ± 20.24			
Marital status	Married	255	99.20	239.25 ± 20.45	–	–	–
	Divorced	1	0.40	–			
	Widowed	1	0.40	–			
Type of family	Nuclear family	142	55.30	239.18 ± 21.51	0.48	0.006 (0.000, 0.025)	0.696
	Stem family	110	42.80	239.37 ± 19.23			
	Step family	2	0.80	227.50 ± 13.44			
	Other	3	1.20	249.67 ± 4.04			
Annual income (in RMB)	<10,0000	36	14.00	231.08 ± 19.66	2.11	0.040 (0.000, 0.085)	0.065
	10,0000–20,0000	90	35.00	239.38 ± 19.59			
	20,0000–30,0000	53	20.60	243.42 ± 17.28			
	300,000–400,000	27	10.50	244.81 ± 19.60			
	400,000–500,000	15	5.80	237.13 ± 15.32			
	>500,000	36	14.00	238.00 ± 27.04			
Work-related changes attributable to disease	Yes	31	12.10	232.58 ± 23.58	−1.97	−0.380 (−0.760, −0.000)	0.050
	No	226	87.90	240.22 ± 19.78			

### 3.2 The scores of FMSCA

The total score on the FMSCA was 239.30 ± 20.38 points. Scores were relatively low in two dimensions, management mindset and family focus, with mean scores of 10.67 ± 4.19 and 10.21 ± 3.32 points, respectively. Scores were higher in the other dimensions, as detailed in Table 3.

**Table 3:** Detailed score of FMSCA (*n*=257).

Subscale	Mean scores of the domains (x ± s)	Mean item scores (x ± s)
Total score	239.30 ± 20.38	29.91 ± 23.81
Children identity	27.41 ± 3.29	4.56 ± 0.21
View of condition	24.42 ± 4.18	4.07 ± 0.47
Management mindset	10.67 ± 4.19	2.67 ± 0.16
Parental mutuality	35.82 ± 5.95	4.48 ± 0.11
Parenting philosophy	23.30 ± 2.55	4.66 ± 0.02
Management approach	85.05 ± 8.32	4.48 ± 0.31
Family focus	10.21 ± 3.32	2.56 ± 1.31
Future expectation	22.41 ± 3.08	4.48 ± 0.13

### 3.3 Univariate analysis of factors influencing the FMSCA

Univariate analysis identified several factors significantly associated with the total score of the FMSCA. Significant differences in total FMSCA scores were observed across nutritional status groups [F (5,251) = 3.12, *η*^2^ = 0.059, 95% CI = (0.010, 0.112), *P* < 0.01]. Levene's test for homogeneity of variances indicated that variances were equal across groups (*P* = 0.121); therefore, Tukey's HSD method was used for *post-hoc* pairwise comparisons. Specifically, children in the wasting group had the highest scores, with a statistically significant difference compared to the obese group (*P* = 0.006) and a marginally significant difference compared to the normal-weight group (*P* = 0.052). The obese group had the lowest scores, but differences with groups other than the wasting group did not reach statistical significance (all *P* > 0.05).

Similarly, the caregiver's educational level significantly influenced the total FMSCA score [F (4,252) = 3.10, *η*^2^ = 0.047, 95%CI = (0.005, 0.095), *P* < 0.05]. Levene's test for homogeneity of variances indicated that variances were equal across the primary caregiver's educational-level groups (*P* = 0.755). Therefore, Tukey's HSD method was employed for *post-hoc* comparisons. The score of the “junior high school or below” group was significantly lower than that of the “junior college” group (*P* = 0.026) and the “undergraduate” group (*P* = 0.010), while no significant differences were observed among the “junior college,” “undergraduate,” “middle school,” and “postgraduate” groups. It is worth noting that statistical comparisons were not conducted for the last two groups in the caregivers’ marriages due to their excessively small sample sizes. Specific findings can be found in Tables 1, 2.

### 3.4 Multivariate linear regression analysis of factors influencing the FMSCA

To identify independent influencing factors, multiple linear regression analysis was performed with the FMSCA score as the dependent variable. Independent variables were selected from those that showed statistical significance in univariate analysis, including the child's nutritional status and the caregiver's educational level. For entry into the regression model, these categorical variables were converted into sets of dummy variables. For nutritional status, the study set the “normal” as the reference categories based on clinical standards. Regarding the educational level of caregivers, the study designated the “junior college” as the reference category according to the baseline classification within China's education system. A stepwise regression approach was employed to construct the model, with inclusion criterion, *P* < 0.05.

The regression model demonstrated overall statistical significance [F (1,253) = 4.47, *P* < 0.001], explaining 8.3% of the variance in the FMSCA total score (*R*^2^ = 0.083, adjusted *R*^2^ = 0.072). Model diagnostics revealed a Durbin-Watson statistic of 1.816, close to 2, indicating no severe autocorrelation in residuals. All variance inflation factors (VIFs) were below the threshold of 5, suggesting the absence of substantial multicollinearity among the independent variables.

As shown in Table 4, compared with “normal”, child's wasting was associated with higher FMSCA scores (Beta = 0.16, *P* < 0.05), indicating better management styles, while child's obesity were associated with lower scores (Beta = −0.13, *P* < 0.05), indicating worse management styles. Regarding educational level, caregivers with junior high school education or below were associated with low scores (Beta = −0.19, *P* < 0.05) compared to those with junior college qualifications, indicating worse management styles. No statistically significant differences in FMSCA scores were observed between other educational groups and the junior college group.

**Table 4:** Multiple linear stepwise regression analysis on influencing factors of the FMSCA (*n*=257).

IV	B	SE	Beta	95%CI		t	*P*
				Lower limit	Upper limit		
Constant	240.64	1.37	–	237.60	243.01	182.69	<0.001
Wasting (vs. normal)	12.61	4.81	0.16	3.14	22.09	2.62	0.009
Obesity (vs. normal)	−11.44	5.41	−0.13	−22.10	−0.78	−2.11	0.035
Junior high school or below (vs. junior college)	−12.57	4.07	−0.19	−20.58	−4.56	−3.09	0.002

B, unstandardized coefficient; CI, confidence interval. Only predictors retained in the final stepwise model (*P* < 0.05) are shown. Model summary: *R*^2^ = 0.08. adjusted *R*^2^ of.07 F(1,253) = 4.47, *P* < .05.

## 4 Discussion

### 4.1 Adaptive family management style in pediatric asthma

This study utilized the FMSCA to evaluate the management styles of children with bronchial asthma within their families. The total scale score was (239.30 ± 20.38), indicating an overall upper-middle level. Among the subscales, scores for the Management Mindset and Family Focus dimensions were relatively low, while the remaining dimensions performed well. This pattern aligns with the adaptive family management style proposed by Knafl et al. (9). It contrasts sharply with the thriving management style reported by Lin et al. (11). Although both types of families demonstrated positive levels across six dimensions, including children identity, view of condition, parental mutuality, parenting philosophy, management approach, and future expectation, the “normalisation” process established by adaptive style families exhibits inherent fragility. This fragility manifests specifically in lower scores on the management mindset and family focus dimensions, indicating that families continue to grapple with persistent challenges of insufficient confidence and maintaining life balance throughout the management process.

The discrepancy is primarily related to disease cognition, access to medical care, and social environment. The children included in this study had relatively long disease durations, enabling primary caregivers to develop considerable disease awareness and acceptance. Additionally, most participants resided in urban areas, with primary caregivers earning over ¥100,000 annually, having access to readily available healthcare resources, and being more likely to have easier access to health-related knowledge. Nevertheless, the long-term management and unstable control of bronchial asthma continue to impose burdens on families. Some caregivers even experienced job changes due to caregiving responsibilities, suggesting that work-care conflicts may further undermine family management effectiveness. Furthermore, caregiver fatigue can arise from misconceptions about corticosteroids (26, 27) and differing disease expectations (28). To address these challenges, it is recommended that nurses lead family meetings involving other household members. Promoting medication knowledge and emphasizing the importance of long-term adherence, while developing flexible family management plans tailored to the child's clinical and cognitive status (29), can improve asthma outcomes while enhancing the family's overall resilience and coping capacity.

### 4.2 Factors influencing family management styles in pediatric asthma

#### 4.2.1 Nutritional status of pediatric patients

The findings indicate that, compared to children of normal weight, those who are wasting children scored higher, while obese children scored lower. The discrepancy may stem from the combined influence of physiological and sociocultural factors. Physiologically, obese children frequently present with elevated serum biomarkerssuch as IL−6, hs-CRP, and TNF-α. These factors may exacerbate asthma control difficulties, thereby impacting family management efficacy (30). Additionally, the pathophysiology of obese children involves fat accumulation compressing the thoracic cavity, impeding lung expansion. This mechanical restriction reduces lung volume and compliance, hinders full airway dilation, and increases airway hyperresponsiveness, thereby heightening susceptibility to asthma attacks (31). Notably, a bidirectional association exists between asthma and obesity, with asthmatic children exhibiting a 23% higher risk of obesity compared to healthy peers (32). This highlights the complex interplay between asthma and obesity, further complicating family management.

Sociocultural factors may also play a significant role. We hypothesize that higher scores among wasting children correlate with compensatory behaviors. Within the Chinese sociocultural context, childhood wasting is often as caregiver neglect, potentially prompting families to adopt more aggressive management strategies to counter societal perceptions. Research indicates that caregivers who are obese themselves may harbor misconceptions about dietary control (33), which similarly influences their attitudes and behaviors toward managing the child's asthma. Future research should further explore how caregivers’ nutritional status influences family management styles. While developing family management plans, one should avoid excessive intervention, adopting a multidisciplinary collaborative model integrating behavioral support strategies (34) is recommended. The approach aims to achieve synergistic management of asthma and weight by improving the family's overall lifestyle.

#### 4.2.2 Educational level of caregivers

The study found that caregivers with junior high school education or less scored lower than those with junior college qualifications, indicating a significant gap in their knowledge and ability to manage disease. This finding matches reports by Fasola et al. (35). Within China's educational framework, junior college education is regarded as the starting point of higher education, signifying that individuals possess a certain capacity for systematic learning and a foundational ability to comprehend information. Caregivers with junior high school education or below exhibit deficiencies in health maintenance awareness and capability, specifically including trouble recognizing asthma attacks early, waiting too long to seek care, not fully reporting symptoms, and misreading medical information (36). These factors together hurt adherence to treatment and limit good care. Therefore, interventions must focus on caregivers with educational level at junior high school level or below. Tiered educational interventions (37) should employ simpler, more accessible language alongside visual educational formats to help caregivers grasp asthma and show them how to care for their children. These steps will boost caregivers’ skills and support them as they adapt to their role.

### 4.3 Limitations

We acknowledge certain limitations in our study. It should be noted that our regression model explained only 8.3% of the variance in family management styles, suggesting that other unmeasured factors likely play important roles. Therefore, our findings should be interpreted as associations rather than causal relationships. Besides, the sample consisted solely of pediatric asthma patients and their caregivers from a single hospital, limiting its representativeness. Data relied on caregiver self-reports, potentially introducing recall bias or social desirability effects that may have compromised the objectivity of management styles assessments. Unmeasured potential confounders, such as home environmental quality, caregiver mental health, and social support systems, were not included, which may have affected the overall interpretation of management styles. Finally, as a cross-sectional study, this research reflects family management style at a specific point in time and cannot reveal how these styles evolve across different stages of the family life cycle. Additionally, the univariate analyses used to screen potential factors did not adjust for multiple comparisons, which may increase the risk of Type I error. Therefore, the identified associations should be interpreted as exploratory and require validation in future confirmatory studies. To overcome the limitations of a single-center design and identify additional intervention targets, conducting large-scale, multi-center studies in the future is crucial.

## 5 Conclusion

In summary, the family management styles of children with bronchial asthma are characterized by an adaptive pattern. The child's nutritional status and the caregiver's educational level are significant factors influencing these management practices. These findings highlight the importance of continuously monitoring of child's nutritional status, alongside providing more targeted guidance and support for caregivers with a junior high school education or below.

## Data Availability

The original contributions presented in the study are included in the article/Supplementary Material, further inquiries can be directed to the corresponding author.
